# Predicting progression and cognitive decline in amyloid-positive patients with Alzheimer’s disease

**DOI:** 10.1186/s13195-021-00886-5

**Published:** 2021-09-06

**Authors:** Hákon Valur Dansson, Lena Stempfle, Hildur Egilsdóttir, Alexander Schliep, Erik Portelius, Kaj Blennow, Henrik Zetterberg, Fredrik D. Johansson, for the Alzheimer’s Disease Neuroimaging Initiative (ADNI)

**Affiliations:** 1 Department of Computer Science and Engineering, Chalmers University of Technology and University of Gothenburg, Gothenburg, Sweden; 2 Department of Psychiatry and Neurochemistry, Institute of Neuroscience and Physiology, The Sahlgrenska Academy at the University of Gothenburg, Mölndal, Sweden; 3 Clinical Neurochemistry Laboratory, Sahlgrenska University Hospital, Mölndal, Sweden; 4 Department of Neurodegenerative Disease, UCL Institute of Neurology, London, UK; 5 UK Dementia Research Institute, UCL, London, UK

**Keywords:** Alzheimer’s disease, Amyloid-beta, Progression, Prediction, Machine learning

## Abstract

**Background:**

In Alzheimer’s disease, amyloid- *β* (A *β*) peptides aggregate in the lowering CSF amyloid levels - a key pathological hallmark of the disease. However, lowered CSF amyloid levels may also be present in cognitively unimpaired elderly individuals. Therefore, it is of great value to explain the variance in disease progression among patients with A *β* pathology.

**Methods:**

A cohort of *n*=2293 participants, of whom *n*=749 were A *β* positive, was selected from the Alzheimer’s Disease Neuroimaging Initiative (ADNI) database to study heterogeneity in disease progression for individuals with A *β* pathology. The analysis used baseline clinical variables including demographics, genetic markers, and neuropsychological data to predict how the cognitive ability and AD diagnosis of subjects progressed using statistical models and machine learning. Due to the relatively low prevalence of A *β* pathology, models fit only to A *β*-positive subjects were compared to models fit to an extended cohort including subjects without established A *β* pathology, adjusting for covariate differences between the cohorts.

**Results:**

A *β* pathology status was determined based on the A *β*_42_/A *β*_40_ ratio. The best predictive model of change in cognitive test scores for A *β*-positive subjects at the 2-year follow-up achieved an *R*^2^ score of 0.388 while the best model predicting adverse changes in diagnosis achieved a weighted *F*_1_ score of 0.791. A *β*-positive subjects declined faster on average than those without A *β* pathology, but the specific level of CSF A *β* was not predictive of progression rate. When predicting cognitive score change 4 years after baseline, the best model achieved an *R*^2^ score of 0.325 and it was found that fitting models to the extended cohort improved performance. Moreover, using all clinical variables outperformed the best model based only on a suite of cognitive test scores which achieved an *R*^2^ score of 0.228.

**Conclusion:**

Our analysis shows that CSF levels of A *β* are not strong predictors of the rate of cognitive decline in A *β*-positive subjects when adjusting for other variables. Baseline assessments of cognitive function accounts for the majority of variance explained in the prediction of 2-year decline but is insufficient for achieving optimal results in longer-term predictions. Predicting changes both in cognitive test scores and in diagnosis provides multiple perspectives of the progression of potential AD subjects.

**Supplementary Information:**

The online version contains supplementary material available at (10.1186/s13195-021-00886-5).

## Background

About 50 million people worldwide suffer from some form of dementia, and 60–80% of all cases have Alzheimer’s disease (AD) [1]. Patients who already suffer from mild cognitive impairment (MCI) are at higher risk of developing AD [2, 3]. Studies have shown that the conversion rate from MCI to AD is between 10 and 15% per year with 80% of these MCI patients progressing to AD after approximately 6 years of follow-up [4, 5]. Identifying those who are at greatest risk of progression to AD is a central problem.

A key pathological hallmark, required for an AD diagnosis, is the accumulation of A *β* peptides into plaques, located extracellularly, and in intracellular tangles, consisting of phosphorylated tau (p-tau) protein [6, 7]. The precipitation of A *β* in the brain appears decades before the patient shows symptoms during the so-called preclinical stage of AD [8–10]. Lower levels of the aggregation-prone peptide A *β*_42_ (or A *β*_42_/A *β*_40_ ratio) together with increased levels of p-tau and total-tau (t-tau) are a core cerebrospinal fluid (CSF) signature of AD [6]. However, despite strong evidence for association between these biomarkers and AD, individuals with significantly lowered A *β* ratio do not necessarily exhibit any cognitive impairment [11, 12]. Therefore, A *β* pathology alone is not sufficient as a predictor of disease progression [13, 14].

Although AD predictors and pathological hallmarks have been researched for many years, today there is still no drug available that cures AD or drastically changes its course. New drug candidates that have potential disease-modifying effects [15] are currently in development and recently, the FDA approved Aduhelm for the treatment of patients with AD under the Accelerated Approval process. The FDA concluded that the benefits of Aduhelm for patients with Alzheimer’s disease outweigh the risks of the therapy.

If a successful treatment is developed, it is of utmost importance that a prognostic tool is available to identify the patients most likely to decline towards AD, to implement preventive treatments and interventions. This leaves the challenge of predicting how patients with A *β* pathology will progress, explaining the variation in cognitive function of such subjects. As a result, a recent focus area in applied statistical and computational research is predicting a change in diagnosis for patients progressing from cognitively normal (CN) to MCI and from MCI to AD [5, 16–19].

Most predictive models of neurodegenerative diseases are based on recent advances in machine learning (ML) models by obtaining data sets with measurements of cognition and neuropathology from large cohorts [16, 20–22]. In this context, classification methods such as random forest [13, 21, 23, 24] and logistic regression (LR) [21, 25–27] have been used to predict whether individuals will decline or remain stable in their diagnosis.

Classification approaches are dependent on the availability of clinical labels and do not focus on capturing patient-specific disease trajectories. To overcome this limitation, disease progression has also been studied with respect to continuous measures of the disease severity [28, 29]. Previous works employed an elastic net linear regression model [30, 31] to predict changes in cognitive test scores to capture the patient’s cognitive ability over time. The most common targets when predicting cognitive decline are the Mini Mental Status Test (MMSE) [32] and the Alzheimer’s Disease Assessment Scale-Cognitive Subscale (ADAS-Cog) [33] scores [34–36].

In prediction modeling, the question arises as to which of the considered input variables are particularly predictive. In addition to predictors of AD diagnosis, relationships between CSF biomarkers (CSF p-tau/A *β*42 ratio and several other biomarkers) and prediction of cognitive decline have been explored [26, 37–39]. However, even though A *β*-positivity has been identified as a strong predictor of disease status, little is known about what determines the disease progression of A *β*-positive subjects [27, 40].

This study aims to predict the future severity of dementia for subjects with established presence of low A *β* levels in CSF. We propose and demonstrate several predictive models of disease progression for three different cohorts, studying two primary aspects of progression: cognitive decline and change in diagnosis. For the former, we predict the change in the MMSE cognitive test score both 2 and 4 years after baseline (the first visit of each patient). For the latter, we use a classification approach to predict whether subjects will have a worse diagnosis 2 years after baseline. Both tasks are addressed using linear and non-linear prediction models, the parameters of which were selected using ML methodology.

A predictive approach could be used to assist healthcare professionals in evaluating and prioritizing patients for treatment. Given that our model builds on only a small set of biomarkers and demographic data, available for most patients, the methodology is widely applicable.

## Methods

### Subjects and ADNI

The data used in this study were obtained from the publicly available Alzheimer’s Disease Neuroimaging Initiative (ADNI) database (http://adni.loni.usc.edu). ADNI collects clinical data, neuroimaging data, genetic data, biological markers, and clinical and neuropsychological assessments from participants at different sites in the USA and Canada to study MCI and AD. Since its inception in 2003, several releases have been made; the cohorts used in this work were assembled from ADNI 1,2,3 and GO.

The compiled data set used in this project includes 2293 subjects that were further filtered by eligibility criteria, such as availability of diagnostic labels and on A *β* ratios. Among the 2293 subjects, there were 749 A *β*-positive subjects. The exclusion flowchart (see Fig. 2) describes how many subjects are assigned to perform a prediction task for the all subjects and A *β*-positive cohorts. For baseline statistics of the processed A *β* cohort, see Table 1. Tables 6 and 7 in the supplementary material show the characteristics of all subjects and A *β*-positive subjects for the three prediction tasks.

**Table 1:** Baseline demographic and clinical characteristics of the ADNI cohort for A *β*-positive subjects and all subjects

		Overall *Aβ*-positive	Missing	CN	MCI	AD	All subjects	All missing
*n*		749		132	411	206	2293	
AGE, mean (SD)		73.7 (7.2)	1	75.2 (6.0)	73.2 (7.0)	73.6 (8.1)	73.2 (7.2)	9
Gender, *n* (%)	m	415 (55.4)	0	55 (41.7)	247 (60.1)	113 (54.9)	1217 (53.2)	5
	f	334 (44.6)		77 (58.3)	164 (39.9)	93 (45.1)	1071 (46.8)	
MMSE, mean (SD)		26.5 (2.8)	0	29.0 (1.2)	27.4 (1.9)	23.3 (2.0)	27.4 (2.66)	5
ADAS13, mean (SD)		20.1 (9.6)	5	10.2 (4.6)	18.4 (6.7)	30.3 (7.7)	17.0 (9.25)	29
TAU, mean (SD)		337.1 (139.2)	7	281.5 (102.5)	334.3 (141.9)	378.6 (141.5)	285.6 (132.9)	1010
ABETA42, mean (SD)		754.0 (320.0)	0	885.3 (396.5)	761.5 (311.6)	654.8 (240.7)	1090.7 (607.5)	1014
FDG, mean (SD)		1.19 (0.15)	157	1.29 (0.11)	1.22 (0.14)	1.06 (0.13)	1.23 (0.15)	806
APOE4, *n* (%)	0	245 (34.5)	39	63 (50.4)	133 (34.5)	49 (24.6)	1162 (54.1)	147
	1	345 (48.6)		56 (44.8)	187 (48.4)	102 (51.3)	780 (36.4)	
	2	120 (16.9)		6 (4.8)	66 (17.1)	48 (24.1)	204 (9.5)	
Hippocampus, mean (SD)		6517.6 (1091.6)	168	7300.8 (788.8)	6585.0 (1029.8)	5883.8 (1008.4)	6794.0 (1185.7)	806
AV45, mean (SD)		1.37 (0.20)	312	1.29 (0.21)	1.36 (0.20)	1.44 (0.18)	1.21 (0.23)	1205
ABETARatio, mean (SD)		0.087 (0.029)	0	0.094 (0.020)	0.086 (0.023)	0.084 (0.023)	0.129 (0.057)	1014

### Determination of amyloid-positive status

**Fig. 1:**
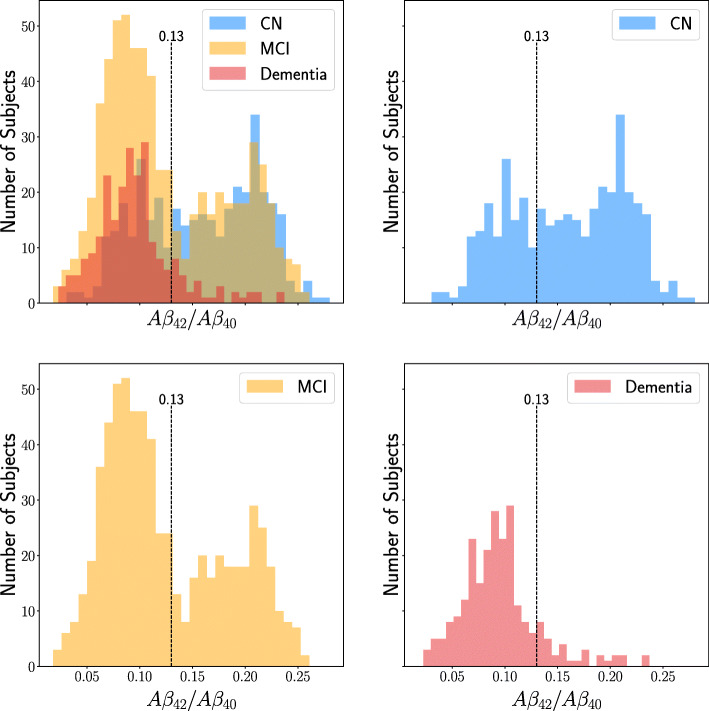
Histogram showing the A *β* ratio of subjects at baseline. The different colored groups represent different diagnoses: dementia, MCI and CN. The upper left histogram shows all diagnoses together and overlaps have blended colors like green and dark red

The presence of A *β* plaques can be detected at a preclinical stage years before the patient shows any symptoms [9, 10]. While A *β* plaques (and tau-levels) may not be the root cause of disease development [41], their abnormal deposits in the brain uniquely define AD [6, 7]. However, even among subjects with A *β* pathology, there is significant variability in symptoms, such as cognitive function. For this reason, our work is focused on predicting progression for subjects with lowered CSF levels of A *β* indicating plaque formations in the brain. Subjects were evaluated for A *β* pathology based on their A *β*_42_/A *β*_40_ ratio (hereinafter simply A *β* ratio) as measured in CSF at baseline. The full cohort was split into three groups: those who had a baseline A *β* ratio lower than 0.13 (A *β*-positive), those who had a higher ratio (A *β*-negative), and those with unknown status. The threshold used in this work is slightly higher than in some other works. For example, a threshold of 0.0975 proposed in [42] for the diagnosis of AD. However, as diagnosing AD was not our primary concern, we let the distribution of ratios themselves decide the threshold, see Fig. 1, rather than tying it to a particular prediction target.

### Progression outcomes

We studied the progression of A *β*-positive subjects with respect to two principal outcomes: change in cognitive function relative to baseline and change in clinical dementia diagnosis.

Cognitive function was assessed using the widely adopted MMSE scale [32]. The MMSE score is commonly used as a target variable in clinical trials analyzing the treatment effects of drugs aimed at enhancing cognition for AD patients and in ML for predicting change in patient’s cognitive ability [43, 44]. The MMSE comprises a series of 20 individual tests covering 11 domains for a total of 30 items. The test covers the person’s orientation to time and place, recall ability, short-term memory, and arithmetic ability. The MMSE score takes values on a scale from 0 to 30 where a lower score represents worse cognitive function [45]. The specific targets of prediction were the changes in MMSE score measured at follow-up visits 2 years after baseline and 4 years, relative to baseline.

Changes in dementia diagnoses were determined by comparing the disease status (CN/MCI/AD) recorded in ADNI at follow-up visits to the status at baseline. For the corresponding prediction task, a binary variable was created, indicating whether or not a subject’s diagnosis had worsened in 2 years. Due to the low number of available subjects after 4 years, changes in diagnosis were evaluated only 2 years after baseline. The models were used to predict whether A *β*-positive subjects would transfer from the CN group at baseline to either MCI or AD or convert from MCI at baseline to AD at a follow-up visit after 2 years.

### Potential predictors

The covariates available at baseline (enrolment in ADNI) contain analyzed biofluid samples from CSF, plasma, and serum including different biochemical-markers such as proteins, hormones, and lipids. Additionally, features extracted from brain imaging biomarkers, such as positron emission tomography (PET) scan and magnetic resonance imaging (MRI) were included. Demographic data such as age and gender were also considered.

The CSF samples include measurements of both A *β*_42_ and A *β*_40_, which are A *β* peptides ending at positions 42 and 40 respectively. Their ratio in CSF measurements has been proposed to better reflect brain amyloid production than their individual measures [46, 47]. Therefore, the ratio A *β* ratio in CSF is calculated and added as a new feature for all subjects with both measurements available.

Predictive models were built on two different sets of features. The first set of features (all features) was preselected following [48] and expanded to include key features from the ADNI TadPole competition [49] in addition to a few features that were available for over 90% of the ADNI cohort. This resulted in a set of 37 features including biomarkers tau, p-tau, and A *β*_42_ in CSF, the PET measures of AV45 and FDG, seven different size measurements of brain regions, and 15 different cognitive tests. Moreover, the FDG-PET data has been measured by a research group of UC Berkeley. The MPRAGEs (Magnetization Prepared Rapid Acquisition Gradient Echo) for each subject is segmented and parcellated with Freesurfer (version 5.3.0) to define a variety of regions of interest in each subject’s native space. The second feature set (cognitive tests only) consists only of the 15 cognitive tests also present in the all feature set. A full list and descriptions of the features are given in Tables 1 and 2 in the supplementary mate-rial. When building models for predicting the change in MMSE score, the MMSE measures at baseline were not included in the predictions since the target output itself was calculated from the change in its baseline value.

### Statistical analyses

We used machine learning methods to train predictive models of cognitive decline within 2 (task A1) and 4 years (task A2) from baseline, as well as a model for predicting worsened diagnosis status (task B) after 2 years. The full procedure, described further below, involved cohort sample splitting and weighting, model selection and fitting, and evaluation.

#### Derivation and evaluation cohorts

**Fig. 2:**
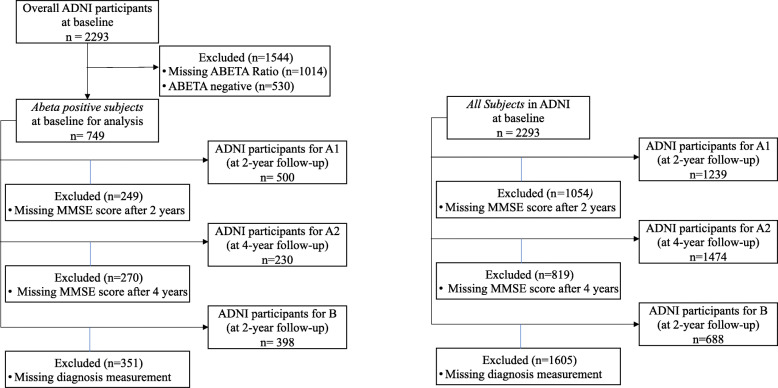
Exclusion flowchart showing the *Aβ*-positive (left) and all subjects (right) cohort. The graphs present the cohorts used for predicting the change in MMSE (A1 and A2) and for the change in diagnosis after 2 years (B)

Due to the small number of A *β*-positive subjects available for each task (500/230/398 for tasks A1/A2/B, respectively), see the exclusion flowchart in Fig. 2), we compared training predictive models from only A *β*-positive subjects to two ways of training using all subjects, irrespective of A *β* status. All models were evaluated only on A *β*-positive subjects, as they are the primary target of this work.

The first derivation setting (A *β* only) used only A *β*-positive subjects for model derivation. This ensures that model parameters are unbiased with respect to the A *β*-positive cohort but may suffer from high variance due to a small sample size. The second setting (all subjects) combined A *β*-positive and A *β*-negative subjects and those without A *β* measurements into one derivation set. Consequently, the derivation sample size has been increased substantially, at the cost of introducing bias into the sample, while the evaluation cohort remains the same.

In the third setting (all subjects, weighted), we applied sample weighting to the all subjects cohort to mimic a larger sample of A *β*-positive subjects. Each subject *i* was assigned a weight *w*_*i*_>0 based on the probability that their individual A *β*-ratio *r*_*i*_ would be observed for an average hypothetical A *β*-positive subject, as estimated using a two-component Gaussian mixture model (GMM) [50] fit to observed ratios.

We let the latent state *C*∈{0,1} of a GMM, fit to the A *β*-ratios of the all subjects cohort, represent A *β*-positivity. The weight *w*_*i*_ was computed as

$$w_{i} = \hat{p}(R=r_{i} \mid C=1)/\hat{p}(R=r_{i})~. $$

This is the ratio of the estimated probability to observe the A *β*-ratio *r*_*i*_ for an average A *β*-positive subject and the overall probability of observing that ratio. This procedure is described further in the supplementary material. The weighting scheme assigns a higher weight to subjects with A *β*-ratio more like that of A *β*-positive subjects and lower to those with higher or unobserved ratios. The weight was clamped between 0.2 and 1.0 so that subjects with unmeasured or very high ratios were given small but non-negligible influence and so that decidedly *Aβ*-positive subjects would be given the weight 1.0. Each prediction model was then fit to the weighted full sample but evaluated only on held-out (unweighted) A *β*-positive subjects.

#### Prediction models and learning objectives

First, we predicted the *change* in MMSE score relative to baseline at the 2-year follow-up (task A1) and 4-year follow-up (task A2) visits using two separate regressions. Second, prediction of change in diagnosis after 2 years (task B) was treated as a binary classification problem (worse diagnosis/not worse diagnosis). For each task, we considered both linear and non-linear estimators.

The first model type used for the MMSE prediction was ordinary least squares linear regression. Similarly, for the classification task, a logistic regression model was used. The second model type used both for regression and classification was tree-based gradient boosting [51]. Gradient boosting is an ensemble method where many weak learners, in our case decision and regression trees, are combined in an iterative fashion to create a strong one. The trees are fit to the negative gradient of the loss function (mean squared error and logistic loss): iteratively, the remaining residual error from the current tree model is the target of the next model. The trained trees are then combined together to form the final model. Our estimates were made using the scikit-learn [52] library.

#### Model selection and evaluation

In this work, we are primarily interested in evaluating how well machine learning models perform for previously unseen subjects. To this end, sample splitting was used to produce an unbiased estimate of the out-of-sample performance of our models. We used *k*-fold cross-validation to divide the A *β*-positive subjects into training and test sets. Selection of hyperparameters for the gradient boosting models then used a nested *k*-fold cross-validation scheme, i.e., cross validation was further performed only on the training samples to select hyperparameters from a grid of possibilities to give a good trade-off between bias and variance.

Cross-validation was used to divide the sample into *k* outer folds of approximately the same size, *k*−1 of which were used for model derivation and 1 for validation. The out-of-sample performance was measured by the average across each combination of *k* derivation and validation folds. In this work, 5-fold cross-validation (*k*=5) was used, training the model on 80% of the data and testing it on the other 20%. This was repeated so that each subset is used exactly once as a validation set and therefore giving a better indication of how well the model performs on unseen data. The overall performance can then be estimated by averaging over the *k* folds [53].

Hyperparameter search was performed within each of the *k* folds; each derivation set was further split again into *k* inner folds *k*−1 of which were used to select a set of model hyperparameters and 1 fold used to validate these. Once the best set was identified, according to the average of the inner held-out folds, the model was retrained on the entire outer derivation fold and tested on the held-out data.

To get a robust and consistent evaluation, this procedure was repeated 10 times for different fivefold cross-validation splits and the average test score given as the final performance, i.e., 50 held-out test score measures from models with (possibly) different hyperparameters are behind the average score and standard deviation reported. As such, it is indicative of the *average* quality we can expect from a model trained on a new similarly-sized sample and evaluated on a held-out similarly sized sample.

The classification models were evaluated using the weighted *F*_1_ score while the regression models used the coefficient of determination—the *R*^2^ score—as a criterion. The *F*_1_ score contained the weighted average of precision and recall. Consequently, this score took both false positives and false negatives into account. The *F*_1_ was chosen since it is usually more useful than accuracy, especially if the data show an uneven class distribution [54]. The *R*^2^ measures how well the independent variables are capable of explaining the variance of the dependent variable and is defined by *R*^2^=1−*S*_*res*_/*S*_*tot*_ where $S_{res} = \sum _{i=1}^{n}(y_{i} - \hat {y}_{i})$ is the residual sum of squares and $S_{tot} = \sum _{i=1}^{n}(y_{i} - \overline {y})$ is the total sum of squares. An *R*^2^ value of 0 indicates that performance is as good as predicting the mean of the variable; higher values are better. This definition of *R*^2^ takes values in [ −*∞*, 1] where negative values represent predictions worse than the mean [55].

## Results

We first report the results of the data preprocessing steps, present cohort statistics, and describe the imputation approach of variables in the ADNI data set. Second, we present the average rates of cognitive decline over time for the CN, MCI, and AD groups, including both A *β*-positive and A *β*-negative subjects. We then inspect the results for models predicting change in MMSE relative to baseline (A1, A2) and change in diagnosis (B). Finally, we study the relationship between predictions in tasks A1 and B with respect to the 2-year follow-ups.

***Preprocessing***

Preprocessing of the data started with zero-mean normalization of continuous variables and one-hot encoding (dichotomization) of categorical variables to reduce variation in the variables’ scales. A simple imputation scheme was used to address missingness in the covariate set. For continuous features, missing values were imputed using mean-imputation while categorical, one-hot encoded features were zero-imputed. These preprocessing steps are performed to maximize the size of the available data and have all features on a similar scale. Since the available cohort for each task was fairly small, and our focus was on held-out prediction risk, which can be estimated in an unbiased way irrespective of the imputation method, model-based imputation was not used. Subjects with missing outcomes were excluded from each corresponding prediction task.

As our main focus is to study the progression of subjects with A *β* pathology, we identified an A *β*-positive cohort by examining the recorded ratio of A *β*_40_ and A *β*_42_ at baseline. To avoid introducing bias in the analysis, the ratio was not imputed. For 1279 subjects, measurements of both A *β*_40_ and A *β*_42_ were available which resulted in an A *β*-positive cohort of *n*=749 subjects (see Fig. 2). It should be noted that, over time, some participants left the study. Consequently, different numbers of subjects were available at follow-ups 2 and 4 years after baseline. The number of A *β*-positive subjects with an MMSE test score available 2 years after baseline was 500 and 230 after 4 years. A total of 398 subjects remained for the diagnosis change prediction task.

The subgroup of A *β*-positive subjects had a mean age of 73.7 years (std. of 7.2) over all the diagnosis groups. The gender distribution over all groups was 55.4% male and 44.6% female. The MMSE score was available for all subjects at baseline: the CN group had a mean value of 29.0 (1.2), the MCI subgroup a mean of 27.4 (1.9), and AD subjects 23.3 (2.0). Another important feature was the tau variable, where measurements were available nearly for all (99%) of the A *β*-positive subjects. Additionally, the main genetic risk factor for AD, the *APOE4* gene, of which a person can have zero, one or two copies, was included for almost all of the A *β*-positive cohort (only 39 were missing) [56].

FDG, measured by positron emission tomography and shown to be a strong marker for AD [47], was absent in 20.9% of the cohort. The statistics of key features used in the three prediction tasks are presented in Table 1 for the subgroup of A *β*-positive subjects and in Table 6 in the supplementary file for the cohort of all subjects.

***Average rates of cognitive decline***

**Fig. 3:**
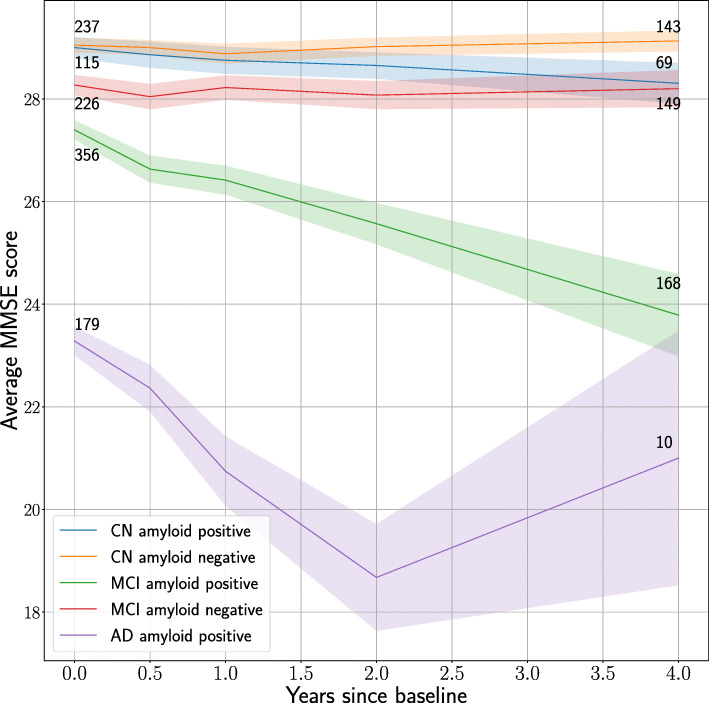
Graph showing the MMSE score development for CN, MCI, and AD subjects split by A *β*-status. The shaded areas represent 95% confidence intervals for the mean values. The number of subjects decreases over time, hence the growing uncertainty bands

For each visit at *t*=1/2,1,2 and 4 years after baseline, the average MMSE score was calculated for observations of different groups divided based on baseline diagnosis (CN, MCI, AD) and A *β*-cohorts (*Aβ*-positives and *Aβ*-negatives). The results are shown in Fig. 3. While we observe a noticeable difference in the rate of cognitive decline between the *Aβ*-positive and negative groups for the MCI subjects, the two CN groups differ only slightly in their trajectories. For the group of AD-positive participants, the mean MMSE score increases again after 2 years. However, it is likely that this change is due to the dropout of a significant number of study participants around this time, resulting in a cohort with different characteristics than at baseline.

The average MMSE score for the A *β*-positive MCI group was 23.79 4 years after the baseline visit, while it was initially 27.40—a decrease on average by 3.61. In contrast, the average score of the MCI A *β*-negative group started at 28.27 and averaging 28.20 score points after 4 years, showing an average decrease of only 0.07. The analysis shows for the CN A *β*-positive and negative groups a decrease in the average score of 0.70 and an increase of 0.08 respectively.

As expected, A *β*-positivity was strongly correlated with faster progression. Although there were remarkable differences in the average deterioration of the MMSE score between the A *β* groups, it should be noted that there was a significant number of missing observations for each group and time point after the baseline visit, due to subjects not undergoing a certain inspection or dropping out of the study. For reference, there were fewer A *β*-positive subjects involved in the study in total (*n*=230) after 4 years than at the beginning of the study (*n*=749) (Fig. 2). The number of participants in the CN A *β*-positive (and negative) groups decreased from 115 and (237) at the beginning of the study to 69 and (143) after 4 years, respectively, while the number of subjects in the MCI A *β*-positive (and negative) groups started at 356 and (226), and declined to 168 and (149) after 4 years. The AD group has a massive drop from 179 at baseline to only 10 subjects after 4 years.

### Task A: Predicting change in MMSE score

In Table 2, we report the performance of the linear regression and the gradient boosting (GB) models that predict the *change* in MMSE scores after 2 and 4 years, respectively, as measured using the average cross-validated *R*^2^ score and standard deviation. The standard deviation was computed across the held-out validation sets corresponding to different cross-validation folds. We compare models fit using only cognitive test scores measured at baseline as predictors, to models fit using a preselected feature set described previously.

**Table 2:** Performance of the linear and gradient boosting regressions, predicting *change in MMSE* 2 and 4 years after baseline for three different cohort selections. We compare models trained on features a) the all features set from baseline and b) from baseline cognitive scores only

*R*^2^ (SD)	2-year follow-up	4-year follow-up
*All features*		
LR, *Aβ* only	0.372 (0.081)	0.205 (0.227)
LR, all subjects	0.354 (0.083)	0.325 (0.134)
LR, all subjects, weighted	0.388 (0.073)	0.304 (0.152)
GB, *Aβ* only	0.287 (0.124)	0.156 (0.244)
GB, all subjects	0.356 (0.108)	0.252 (0.191)
GB, all subjects, weighted	0.338 (0.950)	0.263 (0.192)
*Cognitive tests only*		
LR, *Aβ* only	0.343 (0.087)	0.178 (0.203)
LR, all subjects	0.333 (0.081)	0.228 (0.143)
LR, all subjects, weighted	0.350 (0.079)	0.225 (0.160)
GB, *Aβ* only	0.272 (0.133)	− 0.050 (0.358)
GB, all subjects	0.323 (0.118)	0.149 (0.224)
GB, all subjects, weighted	0.293 (0.114)	0.118 (0.227)

The best 2-year MMSE prediction model achieved an *R*^2^ of 0.388 (std. 0.073) using all features and a linear regression model utilizing all subjects but weighted during training. This model scored marginally higher than restricting the training data to only A *β* subjects with a *R*^2^ of 0.372 (std. 0.081). The gradient boosting models performed worse across the three cohort selections compared with their linear regression counterparts. These results do not indicate any immediate benefit from using nonlinear estimators to model cognitive score change in this sample. The best prediction for the 2-year follow-up using only cognitive tests resulted in an *R*^2^ of 0.350 (std. 0.079) which is only slightly lower than the best model using all features.

The best cross-validated *R*^2^ score for predicting change in MMSE after 4 years was 0.325 (std. 0.134), using all features and a linear regression model using the equally weighted cohort in the training. Using only cognitive tests for this task gives a lower score indicating that other biomarkers offer more than in the 2-year case. Using only the A *β* subjects for this task results in quite poor predictions with high variability compared to utilizing the weighted sample cohort or the weighted equally cohort while training, indicating that more data can significantly improve the training of these models. Similarly to the 2-year setting, the gradient boosting models showed lower performance than the linear models.

Across both tasks A1 and A2, linear models using the larger feature selection and utilizing more subjects than just the cohort containing only A *β* positive subjects performed considerably better in predicting the change of the MMSE score.

**Fig. 4:**
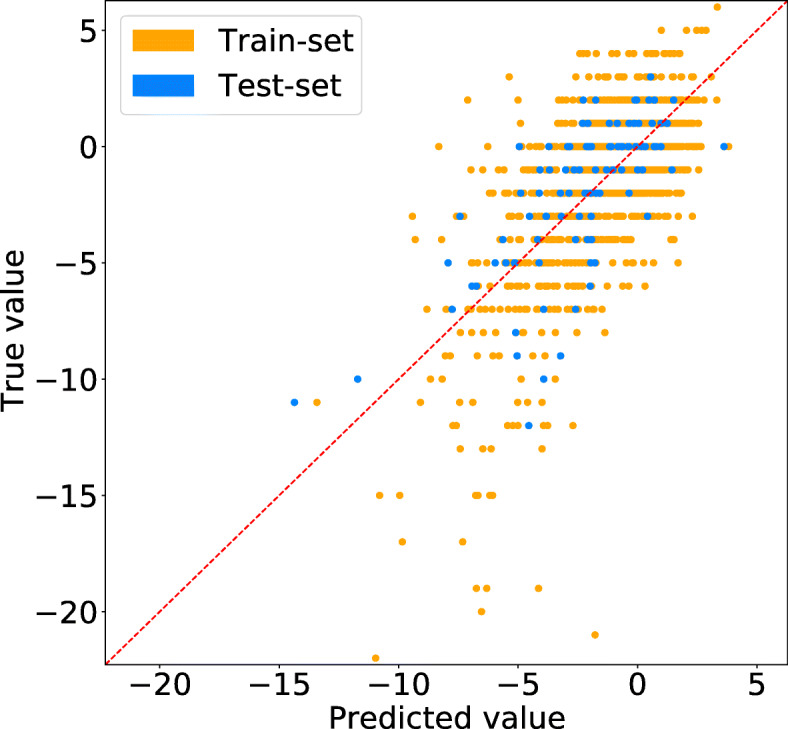
A calibration plot (true vs predicted values) for a linear regression model that predicts the change in MMSE score 2 years from baseline

Figure 4 shows a calibration plot for held-out data corresponding to a single fold from the cross-validation from a linear regression model predicting MMSE change after 2 years. Calibration was good for smaller declines but worse for faster-declining subjects, for which the predictions underestimated the change. This trend was consistent across the two follow-up lengths; there are a few subjects whose change in the MMSE score is significantly larger than others and therefore are more difficult to predict. These outliers may potentially also have decreased the quality of predictions of other data points.

In Table 4 in the supplementary material, we list the importance measures of features across the 2-year prediction models using all features. For predicting change in the MMSE score, the most important features were baseline cognitive scores, with ADAS13, TRABSCORE, and ADAS11 being the most predictive. The linear models additionally selected the mPAACCtrailsB, LDELTOTAL and ADASQ4 while other cognitive tests such as FAQ and RAVLT_immediate were chosen by the gradient boosting models as part of the most predictive features. This is expected since subjects with early disease status (e.g., with high baseline MMSE score) tend to change less rapidly than already progressing subjects [57]. For this reason, we included also the results of estimators predicting change in MMSE based only on baseline cognitive scores in Table 2. However, we see that across all models and tasks, the performance improved slightly by using additional predictors.

Several features were only identified as important by one or two models across the cohorts. For instance, the volume measurement of WholeBrain was selected by two gradient boosting models including all subjects equally weighted and the A *β* only cohort. Moreover, the FDG feature, obtained by PET and known to be a strong marker for AD [47] is selected in the cohort including only A *β* positives among the five most important features.

The estimated levels of A *β* measured through A *β*_42_ in CSF and AV45 PET scans showed low predictive power in the context of other features across all cohorts and models. For example, the A *β*_42_ measurements were only included with a coefficient of 0.30 in the linear regression model using all subjects equally weighted and −0.01 when training with only A *β* subjects and the AV45 is rated even less predictive.

For the 4-year predictions, the features that are rated most important in the linear regression models are a dementia diagnosis, TAU and PTAU proteins in CSF followed by the mPACCtrailsB and ADASQ cognitive tests. The gradient boosting models however deem FDG along with the cognitive scores ADAS13, FAQ, and mPACCtrailsB to be of most importance for making predictions. Comparing to the 2-year predictions, it is interesting to see the increased value in using biomarkers other than cognitive tests. The 4-year predictions also indicate low predictability by A *β*-related features when predicting the rate of decline in A *β*-positive individuals.

### Task B: Predicting diagnosis change

In Table 3, we report the results of predicting a worsened diagnosis at the 2-year follow-up visit. Gradient boosting using all features and an equally weighted cohort during training resulted in the best performance, achieving a cross-validated weighted *F*_1_ score of 0.791 with a standard deviation of 0.042. However, the gradient boosting model with weighted subjects in the cohort reaches only a slightly lower weighted *F*_1_ score of 0.782 with a standard deviation of 0.040. The logistic regression models consistently perform worse than the gradient boosting ones on the diagnosis prediction for the 2-year follow-up.

**Table 3:** Performance of the classification models in predicting *change in diagnosis* 2 years after baseline for three different cohort selections

Weighted *F*_1_ (SD)	Follow-up after 2 years	
	All features	Cognitive tests only
LR, *Aβ* only	0.763 (0.050)	0.761 (0.046)
LR, all subjects	0.781 (0.044)	0.762 (0.050)
LR, all subjects, weighted	0.776 (0.047)	0.770 (0.046)
GB, *Aβ* only	0.770 (0.043)	0.784 (0.041)
GB, all subjects	0.788 (0.045)	0.793 (0.039)
GB, all subjects, weighted	0.786 (0.046)	0.787 (0.037)

When using only the cognitive tests, the best performing model also uses gradient boosting and a cohort including all subjects weighted equally achieving a weighted *F*_1_ score of 0.787 with a standard deviation of 0.043. This is very close to the previous result using all features. Similarly, the models using only cognitive tests performed marginally worse than their counterparts using all features. In summary, additional features lead to only a slight improvement in the performance for both logistic regression and gradient boosting.

The most important features for the diagnosis models over all three training cohorts are LDELTOTAL and mPACCtrailsB. This result demonstrates that the two most important features in progression prediction belong to the group of cognitive assessment. The logistic regression models also selected as important: TAU, PTau, two *APOE4* genes, and *DX*_*NUM*_1.0 which represents the MCI diagnosis at baseline. However, the gradient boosting models identified several other cognitive test scores as important features, for example, FAQ, TRABSCOR, and ADAS13. Similarly, to the prediction of the change of MMSE score, one can conclude that the A *β*_42_ obtained by CSF as well as the AV45 retrieved by PET are not among the most important features for any of the diagnosis change models.

We can conclude that the logistic regression models and the gradient boosting models rely on similar features. There are bigger differences between important features in logistic regression models than those using gradient boosting.

### Relating predicted cognitive decline & diagnosis change

**Fig. 5:**
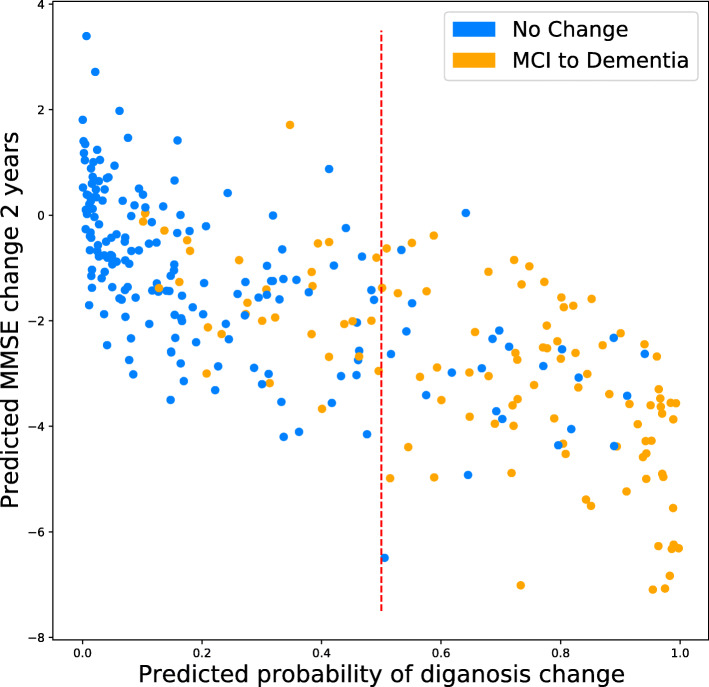
Predicted change in MMSE score and the predicted probability of a change in diagnosis after 2 years in the baseline-MCI group. Points are color-labeled based on their observed change in diagnosis

In Fig. 5, we plot the predictions made by models for tasks A1 and B for the same set of baseline-MCI subjects. Overall, we see a strong correlation between predicted cognitive decline (negative change in MMSE) and predicted change from MCI to AD status. The variance in predicted MMSE change is larger for AD-transitioning subjects than for MCI-stable subjects.

## Discussion

Formation of amyloid-beta plaques in the brain is a hallmark of Alzheimer’s disease. Only recently, the first drug which may mitigate or slow down the formation of these plaques was approved by the FDA [58, 59]. To best target future interventions of this kind, it is of great interest to identify individuals who are most likely to suffer rapid cognitive decline. Since presence of A *β* plaques is required for an AD diagnosis and can be detected early in CSF and plasma, successful prediction of who among A *β*-positive subjects are likely to deteriorate first could have significant clinical implications.

Machine learning approaches, including classification [23, 24] and regression [26, 28] methods, have been used to predict progression of patients from CN to MCI and from MCI to AD. The results show that subjects who already have cognitively declined are most likely to deteriorate more rapidly. However, although such studies have shown that A *β* levels among others are strong predictors of the transition from MCI to an AD diagnosis [13, 20, 27], prediction of progression specifically for patients with established amyloid pathology is so far unexplored.

In this work, we studied prediction of cognitive decline in an A *β*-positive cohort using machine learning methods. We applied multivariate statistical analyses to explain the variation in changes in cognitive scores and diagnoses, between subjects in the ADNI dataset, as a function of commonly available clinical variables. We found that the predictability of changes in cognitive test scores was low, leaving a large portion of variance unexplained. Our results complement previous works which show good discrimination of progressing and non-progressing subjects [16, 21] in cohorts comprising both A *β*-positive and A *β*-negative subjects. In particular, we show that discriminating between subjects who are potential candidates for drugs designed to reverse or slow down A *β* plaque formation presents a harder prediction task.

### Predictors of progression in amyloid-positive subjects

Confirming previous results, we found that the ratio of A *β*_42_ and A *β*_40_ CSF level is a good first-line predictor of decline in the MMSE score [43]. However, when limiting the cohort to only the A *β*-positive subjects, the predictive power of the levels of A *β*_42_ and A *β*_40_ was substantially reduced. In other words, the A *β* biomarkers served predominantly to produce a binary grouping of subjects.

The most important features for predicting disease progression in all considered tasks were baseline cognitive test scores. Although related work has not focused specifically on the A *β*-positive cohort, these results are consistent with previous results in selecting cognitive tests such as the MMSE and ADAS13 tests as important predictive features [29, 44]. Our analysis demonstrated that cognitive test results indicate well how the individual will progress and that those who were already cognitively impaired would likely deteriorate more. Since most of the cognitive test scores are highly correlated, several cognitive scores could perhaps be combined and summarized in a joint variable rather than using all of them separately. Apart from cognitive scores, some of the CSF biomarkers, brain scans and other biomarkers showed lower average importance as predictors for progression when including all subjects. This can partially be explained due to the higher missingness of these features when viewing all subjects.

### Increasing training cohort

Increasing the number of subjects by adding those that were not in the A *β*-positive cohort to the training set consistently increased the predictions performance for that group. Therefore, it seems the A *β*-negative subjects have fairly similar characteristics that determine their cognitive decline. A weighting procedure allowing us to include more subjects in the training gave a better performance than using only the subjects we were interested in predicting. The increased performance from the addition of out-of-cohort samples also indicates that more data would increase the quality of the prediction tasks even further. In the case of predicting MMSE change after 4 years, using a small cohort of only A *β*-positive subjects gave a drastically worse performance.

### MMSE as target variable

The MMSE score has been used frequently in dementia research for grading the cognitive state of patients [60, 61]. For this reason, the change in MMSE score was used in this work as a target variable and thus as a proxy for a person’s cognitive change. The test benefits from high practicability as the typical administration time is only 8 min for cognitively unimpaired individuals and increases to 15 min for individuals with dementia. Internal consistency appears to be moderate and test-retest reliability good [62].

The MMSE is neither the most accurate nor the most efficient instrument for assessing cognitive impairment, nor is it designed specifically for AD. Despite its frequent use, the MMSE lacks sensitivity in patients with high levels of premorbid education and suspected early impairment [63]. Especially for studies that screen cognitively normal populations for evidence of cognitive impairment, the Montreal Cognitive Assessment (MOCA) may be better able to detect age-related cognitive decline in adults since it eliminates the ceiling effects of MMSE [64]. The ADAS13 cognitive test which we used in the primitive studies could also function as a target variable. The ADAS13 test is also commonly used in clinical trials to thoroughly identify incremental improvements or deteriorations in cognitive performance. Although the ADAS is genuinely accurate in distinguishing individuals with normal cognition from those with impaired cognition, some research studies indicate that the ADAS test may not be difficult enough to consistently detect only mild cognitive impairment [33, 65, 66]. Alternatively, for future work, the outcome variable could be a combination of several cognitive tests, which outweighs the individual characteristics of a single cognitive test.

### Clinical implications

Prediction of cognitive decline among A *β*-positive subjects could have clinical implications in a scenario where a disease-modifying drug becomes available on the market. In this case, our approach could be used to assess how an A *β*-positive person, either unimpaired or already in cognitive decline, might develop in the near future. With a further developed predictive approach, physicians could be supported in the prioritization and evaluation of patients for treatment. In particular, models with interpretability aspects may encourage clinicians to use machine learning-based decision-making methods in a clinical context. Further, our approach benefits from relying only on a small number of biomarkers and demographic data that are widely available for many patients and therefore provides high practical relevance. In order to be able to generalize results even better, more accessible patient data will be needed in the future. For an efficient, timely, and practical approach to predicting disease development in Alzheimer’s patients, the approach of precision medicine could be important. With the goal of improving the health of well-defined patient populations, precision medicine will affect all stakeholders in the healthcare system at multiple levels, from the individual perspective to the societal perspective [67].

## Limitations

Our study should be viewed in light of the following limitations. First, there was significant missingness in the target outcome variables, MMSE and diagnosis status, for all prediction tasks. Since these are the targets of prediction, they were not imputed and only subjects with the available output variables were included. Consequently, the cohorts for tasks A1, A2, and B were all different and potentially biased subsets of the initial cohort. For example, the cohort sizes for the regression tasks differ based on whether the MMSE test score variable was available after 2 years (A1, *n*=500) or after 4 years (A2, *n*=230).

The missingness of outcome variables at follow-up time is partly explained by subjects leaving the study before follow-up. The reason for subjects to end their participation in the study is not known but may be related to disease progression [68]. This phenomenon can bias the trend of the A *β* positive subjects decreasing their MMSE score (Fig. 3). However, the dropout rate of people was around 40% in both CN groups and the MCI A *β*-negative one while there was more dropout in the MCI A *β*-positive group where it was 55% and a staggering 94% for the AD group. Consequently, if more people with lower cognitive function would have been included, the average MMSE score would be lower, and therefore, the slope of the graph would be slightly steeper and result in an even lower average MMSE score.

As a consequence of the prohibitively small and imbalanced cohorts, we performed a grouped analysis. The use of non- *Aβ*-positive subjects in deriving progression prediction models reduces variance by increasing the sample size of cohorts that had small numbers of subjects. However, this risks bias in terms of the best model for *Aβ*-positive subjects. Note that *Aβ*-positive-negative subjects were used in the derivation of predictive models, but not in evaluation.

## Conclusions

We studied the problem of predicting disease progression and cognitive decline of potential AD patients with established A *β* pathology in the ADNI database. The best performing model achieved a performance of *R*^2^=0.388 predicting the change in MMSE scores 2 years after baseline using a linear regression model based on a cohort with weighted samples in the training cohort using all features at baseline. Similarly, a gradient boosting model with all subjects weighted equally predicted the change in diagnosis with high accuracy (*F*_1_=0.791) when using all features. For the most accurate predictions, our models combine variables measured at the baseline such as cognitive tests, CSF biomarkers, proteins and genetic markers. Among these, baseline cognitive tests scores were found to be the strongest predictors, accounting for most of the variance explained by all features, across models. Finally, we identified that even though the A *β*_42_/A *β*_40_ ratio is a good predictor for AD in the preclinical phase, the respective levels of A *β* are less useful in predicting progression among only A *β*-positive subjects.

## Supplementary Information

**Additional file 1** The supplementary material includes a method describing weighting of cohorts, along with lists of the cognitive tests and other features. Hyperparameter values, and two tables showing the feature importance for 2 and 4 years after baseline are presented.

## Data Availability

I can confirm I have included a statement regarding data and material availability in the declaration section of my manuscript.

## Abbreviations

AD: Alzheimer’s disease dementia;
ADAS-Cog: Alzheimer’s Disease Assessment Scale-Cognitive Subscale
ADNI: Alzheimer’s Disease Neuroimaging Initiative
A *β*: Amyloid- *β*
A *β* ratio: A *β*_42_/A *β*_40_ ratio
A *β*-positive: A *β* ratio lower than 0.13
A *β*-negative: A *β* ratio higher than 0.13
A1: Predicting cognitive decline after 2 years
A2: Predicting cognitive decline after 4 years
B: Predicting worsened diagnosis status
CN: Cognitively normal
CSF: Cerebrospinal fluid
EMA: European Medicines Agency
FDA: Food and Drug Administration
GB: Gradient boosting
GMM: Gaussian mixture model
LR: Logistc regression
MCI: Mild cognitive impairment
ML: Machine learning
MMSE: Mini Mental Status Test
MRI: Magnetic resonance imaging
PET: Positron emission tomography
std: Standard deviation
