# Supplementary material for: Predicting progression and cognitive decline in amyloid-positive patients with Alzheimer’s disease
Source: Alzheimers Res Ther. 2021 Sep 6;13:151. doi: 10.1186/s13195-021-00886-5 (PMC8422748; doi:10.1186/s13195-021-00886-5)
Supplement: Supplementary file 1 — Additional file 1 The supplementary material includes a method describing weighting of cohorts, along with lists of the cognitive tests and other features. Hyperparameter values, and two tables showing the feature importance for 2 and 4 years after baseline are presented. [file 13195_2021_886_MOESM1_ESM.pdf]

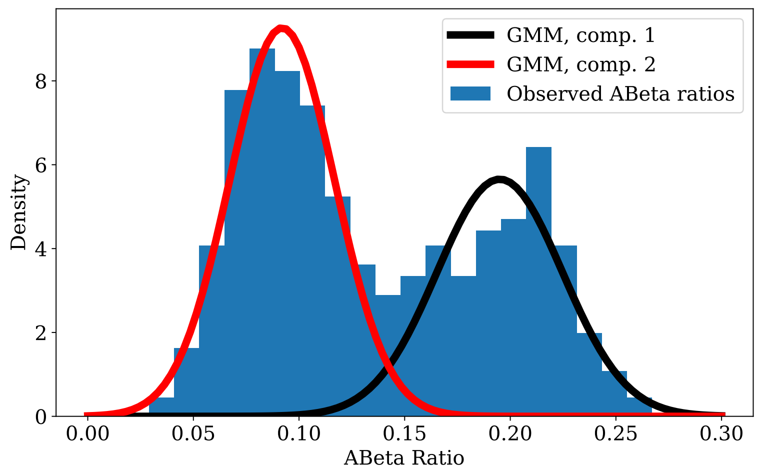

Figure 1 Observed Aβ-ratio and the two components of a Gaussian Mixture Model (GMM) fit to these.

1 Weighting of non-Aβ-positive subjects

Making use of non-Aβ-positive subjects in the derivation of progression prediction models reduces variance by increasing the sample size but introduces potential bias in the results. To trade off between bias and variance, we may weight subjects so that the weighted population more closely resembles the population of Aβ-positive subjects. We use as basis for this weighting the Aβ-ratio itself, here denoted R. As is well known, the Aβ-ratio is well modeled by a mixture of two normal distributions, one component corresponding to Aβ-positive subjects and one corresponding to Aβ-negative ones, see Figure 1. By interpreting one component as representing Aβ-positive subjects, we can weight subjects based on the probability that their ratio would be observed under that component. Let $C = 1$ indicate that a subject is Aβ-positive in the GMM (the ratio was observed from the positive component). Specifically, we define a weight for subjects with observed ratio r as,

$$w(r) = \frac{p(R = r \mid C = 1)}{p(R = r)} \ .$$

By weighting each sample i with $w_i = w(r_i)$ when fitting predictive models, the bias of the models will be reduced compared to the unweighted extended sample, and the variance will be reduced compared to models fit to only Aβ-positive subjects. As the two components are so separated, and the ratio is unobserved for some subjects, we clamp weights between 0.2 and 1.0 by defining, with $\epsilon = 0.2$

$$\tilde{w}_i = \begin{cases} w(r_i)(1-\epsilon) + \epsilon, & \text{if } r_i \text{ observed} \\ \epsilon, & \text{if } r_i \text{ not observed} \end{cases} \ .$$

2 List of cognitive tests

Table 1 List of cognitive tests identified this work as strong predictors for all three models. List shows abbreviations used, name in ADNI database and explanation

Abbreviation	Name in database	Explanation
ADAS11	ADAS11	The Alzheimer's Disease Assessment Scale–Cognitive Subscale, 11 item version. A cognitive test score.
ADAS13	ADAS13	The Alzheimer's Disease Assessment Scale–Cognitive Subscale, 13 item version. A cognitive test score.
ADASQ4	ADASQ4	ADAS Delayed Word Recall. A cognitive test score.
CDRSB	CDRSB	Clinical Dementia Rating Scale–Sum 2253 of Boxes. A cognitive test score.
FAQ	FAQ	Functional Activities Questionnaire. A cognitive test score.
LDELTOTAL	LDELTOTAL	Logical Memory - Delayed Recall. A 2248 cognitive test score.
MMSE	MMSE	Mini Mental State Examination. A cognitive test score.
mPACCdigit	mPACCdigit	ADNI modified Preclinical Alzheimer's Cognitive Composite (PACC) with Digit Symbol Substitution. A cognitive test score.
mPACCtrails	mPACCtrails	ADNI modified Preclinical Alzheimer's Cognitive Composite (PACC) with Trails B. A cognitive test score.
MOCA	MOCA	Montreal Cognitive Assessment (MoCA) Test for Dementia. A cognitive test score.
RAVLT I	RAVLT$_{immediate}$	Rey's Auditory Verbal Learning Test (RAVLT) Immediate (sum of 5 trials). A cognitive test score.
RAVLT L	RAVLT$_{learning}$	Rey's Auditory Verbal Learning Test (RAVLT) Learning. A cognitive test score.
RAVLT F	RAVLT$_{forgetting}$	Rey's Auditory Verbal Learning Test (RAVLT) Forgetting (trial 5 - delayed). A cognitive test score.
RAVLT P F	RAVLT$_{perc-forgetting}$	Rey's Auditory Verbal Learning Test (RAVLT) Percent Forgetting. A cognitive test score.
TRABSCOR	TRABSCOR	Trail-making test B. A cognitive test score.

3 List of other features

Table 2 List of features that are not cognitive tests. Shows abbreviations used, name in ADNI database and explanation

Abbreviation	Name in database	Explanation
ABETA42	ABETA	Amyloid β 1-42 measured from CSF.
FDG	FDG	Average FDG-PET of angular, temporal, and posterior cingulate
Ventricles	Ventricles	San Fransisco (UCSF) ventricles size
Hippocampus	Hippocampus	San Fransisco (UCSF) hippocampus size
WholeBrain	WholeBrain	San Fransisco (UCSF) whole brain size
Entorhinal	Entorhinal	San Fransisco (UCSF) entorhinal size
Fusiform	Fusiform	San Fransisco (UCSF) fusiform size
MidTemp	MidTemp	San Fransisco (UCSF) Middle temporal size
TAU	TAU	Total tau protein measured from CSF
PTAU	PTAU	Phosphorylated tau protein measured from CSF.
DX_NUM_1.0	DX	Categorical value indicating the diagnosis of MCI
DX_NUM_2.0	DX	Categorical value indicating the diagnosis of AD
ICV	ICV	Intracranial volume
AV45	AV45	Average AV45 SUVR of frontal, anterior cingulate, precuneus, and parietal cortex relative to the cerebellum
AGE	AGE	Age of subject
VSBPDIA	VSBPDIA	Seated Blood Pressure: Diastolic
VSBPSYS	VSBPSYS	Seated Blood Pressure: Systolic
VSPULSE	VSPULSE	Seated Pulse Rate
VSWEIGHT	VSWEIGHT	Weight of subject
Gender_1.0	PTGENDER	Categorical value indicating the gender male
APOE4_1.0	APOE4	Categorical value indicating having one APOE4 alleles
APOE4_2.0	APOE4	Categorical value indicating having two APOE4 alleles

4 Hyperparameters tested for gradient boosting

Table 3 Model parameters used in grid search for the gradient boosting models. Other values were set as the default values.

Parameter	Regression	Classification
No. of estimators	100, 200	100, 200
min. samples leaf	1	10
Max. depth	2,3,5	None, 2,3,5
Min. samples needed to split a node	8,16,24,36	8,16,24,36

5 Tables of feature importance

Table 4 Measures of how important features are to the output predictions after two years. The feature importance is given as the linear coefficients for linear models and how often a feature is used for splitting trees in gradient boosting. The five highest absolute values for each column are colored green. The columns are different models used.

Task	2-year MMSE change						2-year diagnosis change					
Esitmator	Linear regression			Gradient boosting			Logistic regression			Gradient boosting		
Cohort	All Subjects, Weighted	$A\beta$ Only	All Subjects	All Subjects, Weighted	$A\beta$ Only	All Subjects	All Subjects, Weighted	$A\beta$ Only	All Subjects	All Subjects, Weighted	$A\beta$ Only	All Subjects
ADAS13	-1.61	0.12	-3.99	0.22	0.16	0.19	-0.02	-0.45	0.50	0.04	0.04	0.04
TRABSCOR	-1.06	-1.18	-1.01	0.08	0.08	0.08	0.56	0.37	1.13	0.04	0.04	0.03
DX_NUM_1.0	0.50	0.31	0.68	0.00	0.00	0.00	-2.29	-2.16	-2.62	0.00	0.00	0.00
TAU	-0.41	-0.06	-0.98	0.01	0.01	0.01	1.27	1.44	2.00	0.04	0.03	0.02
mPACCtrailsB	-2.57	-2.50	-3.07	0.03	0.02	0.04	1.21	-0.13	3.66	0.22	0.21	0.26
APOE4_2.0	-0.47	-0.64	-0.31	0.00	0.00	0.00	1.17	0.83	1.35	0.01	0.00	0.01
LDELTOTAL	0.94	0.82	0.92	0.03	0.04	0.02	-1.15	-0.67	-2.01	0.10	0.07	0.10
ADAS11	0.03	-1.07	1.47	0.14	0.18	0.18	0.14	0.43	-0.13	0.01	0.02	0.01
PTAU	0.29	0.00	0.67	0.02	0.02	0.01	-0.94	-1.16	-1.44	0.03	0.03	0.02
WholeBrain	0.18	0.07	0.43	0.04	0.06	0.02	-0.02	-0.23	0.10	0.01	0.01	0.01
RAVLT_immediate	0.36	0.32	0.26	0.05	0.02	0.09	-0.71	-0.77	-0.79	0.04	0.04	0.06
ADASQ4	-0.31	-0.99	0.69	0.01	0.00	0.01	0.60	0.77	0.73	0.00	0.00	0.01
FAQ	-0.36	-0.53	-0.22	0.04	0.02	0.05	0.50	0.42	0.68	0.10	0.09	0.10
FDG	0.22	0.23	0.24	0.04	0.04	0.04	-0.20	-0.11	-0.38	0.04	0.04	0.03
MMSE	-	-	-	-	-	-	-0.74	-0.34	-1.64	0.00	0.00	0.00
Gender_1.0	-0.37	-0.26	-0.57	0.00	0.00	0.00	-0.37	-0.40	-0.27	0.00	0.00	0.00
CDRSB	-0.44	-0.22	-0.63	0.01	0.02	0.00	0.35	0.37	0.32	0.03	0.03	0.04
APOE4_1.0	0.13	-0.12	0.33	0.00	0.00	0.00	0.35	-0.03	0.64	0.00	0.00	0.00
ABETA42	0.13	-0.01	0.30	0.02	0.03	0.01	-0.33	-0.17	-0.42	0.02	0.02	0.01
mPACCdigit	-0.18	-0.27	0.44	0.03	0.02	0.04	0.32	0.74	-0.10	0.03	0.04	0.02
Ventricles	-0.17	0.06	-0.36	0.02	0.03	0.01	0.29	0.21	0.38	0.02	0.02	0.02
MidTemp	0.30	0.28	0.29	0.02	0.03	0.02	-0.27	-0.14	-0.38	0.02	0.03	0.02
Entorhinal	-0.13	-0.09	-0.15	0.01	0.01	0.02	-0.24	-0.21	-0.30	0.03	0.03	0.02
AV45	-0.07	-0.01	-0.15	0.01	0.01	0.01	0.23	0.27	0.33	0.01	0.01	0.01
RAVLT_learning	-0.19	-0.24	-0.16	0.00	0.00	0.01	0.22	0.12	0.19	0.01	0.01	0.01
VSPULSE	0.12	0.09	0.14	0.01	0.03	0.01	0.20	0.17	0.25	0.01	0.01	0.01
AGE	0.43	0.40	0.44	0.03	0.02	0.04	-0.18	-0.38	-0.06	0.02	0.02	0.02
RAVLT_forgetting	0.21	0.52	0.07	0.02	0.01	0.01	0.14	0.37	0.08	0.01	0.01	0.01
VSWEIGHT	0.14	0.04	0.21	0.02	0.02	0.01	-0.14	-0.19	-0.15	0.01	0.01	0.01
MOCA	0.15	0.16	0.13	0.01	0.00	0.01	-0.12	-0.16	-0.08	0.00	0.00	0.00
Fusiform	0.14	0.12	0.27	0.02	0.03	0.01	-0.09	-0.05	-0.10	0.02	0.01	0.02
VSBPDIA	0.20	0.09	0.28	0.02	0.02	0.01	-0.06	0.03	-0.20	0.01	0.01	0.00
VSBPSYS	0.06	-0.02	0.17	0.01	0.01	0.01	-0.06	-0.10	0.02	0.01	0.02	0.01
Hippocampus	-0.05	-0.03	-0.00	0.02	0.02	0.01	0.03	-0.08	0.13	0.02	0.03	0.03
ICV	-0.28	-0.21	-0.57	0.01	0.01	0.02	0.02	0.13	-0.06	0.02	0.03	0.01
RAVLT_perc_forgetting	-0.11	-0.57	0.13	0.01	0.00	0.01	-0.00	-0.22	0.07	0.02	0.02	0.02
DX_NUM_2.0	0.47	0.03	0.86	0.00	0.00	0.00	-	-	-	-	-	-

Table 5 Measures of how important features are to the output predictions after four years. The feature importance is given as the linear coefficients for linear models and how often a feature is used for splitting trees in gradient boosting. The five highest absolute values for each column are colored green. The columns are different models used.

Task	4-year MMSE change					
Esitmator	Linear regression			Gradient boosting		
Cohort	All Subjects, Weighted	$A\beta$ Only	All Subjects	All Subjects, Weighted	$A\beta$ Only	All Subjects
DX_NUM_2.0	2.67	1.52	3.37	0.00	0.00	0.00
TAU	-1.85	-1.13	-3.99	0.02	0.01	0.01
mPACCtrailsB	-1.70	-3.21	-0.02	0.06	0.02	0.10
PTAU	1.48	0.72	3.57	0.02	0.01	0.02
ADAS13	-0.54	1.07	-2.28	0.19	0.11	0.18
ADAS11	-0.54	-1.44	0.39	0.03	0.05	0.02
ADASQ4	-1.11	-1.93	-0.28	0.05	0.05	0.04
FDG	0.75	0.43	1.11	0.16	0.23	0.10
FAQ	-0.19	-0.28	-0.02	0.06	0.07	0.06
TRABSCOR	-1.00	-1.51	-0.58	0.03	0.02	0.06
mPACCdigit	-0.57	0.37	-1.63	0.02	0.01	0.03
LDELTOTAL	0.99	1.22	0.62	0.02	0.03	0.03
RAVLT_perc_forgetting	-0.89	-0.87	-0.62	0.00	0.01	0.01
RAVLT_forgetting	0.88	0.85	0.78	0.01	0.01	0.01
AGE	0.69	0.53	0.96	0.02	0.03	0.02
DX_NUM_1.0	0.69	0.69	0.38	0.00	0.00	0.00
WholeBrain	-0.56	-0.07	-1.06	0.01	0.01	0.01
Gender_1.0	-0.50	-0.56	-0.55	0.00	0.00	0.00
APOE4_2.0	-0.45	-0.92	0.15	0.00	0.00	0.00
CDRSB	-0.43	-0.32	-0.60	0.01	0.02	0.01
MidTemp	0.36	0.30	0.33	0.01	0.01	0.02
AV45	-0.36	-0.27	-0.66	0.03	0.02	0.03
Fusiform	0.34	0.13	0.68	0.01	0.02	0.02
ABETA42	0.31	0.18	0.48	0.03	0.02	0.03
VSPULSE	0.25	0.09	0.39	0.01	0.01	0.00
VSWEIGHT	0.23	0.05	0.36	0.04	0.04	0.03
Hippocampus	0.20	0.22	0.30	0.02	0.02	0.03
RAVLT$_learning$	-0.18	-0.12	-0.18	0.03	0.04	0.02
ICV	-0.17	-0.29	-0.22	0.01	0.02	0.01
Entorhinal	0.14	0.07	0.27	0.02	0.02	0.02
APOE4_1.0	-0.12	-0.11	-0.27	0.00	0.00	0.00
RAVLT_immediate	-0.10	0.10	-0.33	0.03	0.03	0.05
VSBPDIA	0.09	0.08	0.13	0.01	0.01	0.01
MOCA	-0.06	-0.15	0.02	0.00	0.00	0.00
Ventricles	-0.04	-0.05	0.05	0.02	0.02	0.02
VSBPSYS	-0.02	-0.09	-0.04	0.01	0.01	0.01

Table 6 [Follow-up demographic and clinical characteristics of the ADNI cohort for *All Subjects* for the three prediction tasks.]

		Change in diagnosis 2 years	Change MMSE 2 years	Change MMSE 4 years	Complete cohort
n		1239	1474	688	2293
AGE, mean (SD)		73.17 (6.88)	73.33 (7.07)	72.87 (6.77)	73.21 (7.22)
Gender n (%)	m	661 (53.3)	786 (53.3)	378 (54.9)	1217 (53.2)
	f	578 (46.7)	688 (46.7)	310 (45.1)	1071 (46.8)
MMSE, mean (SD)		28.30 (1.69)	27.70 (2.41)	28.32 (1.65)	27.36 (2.66)
ADAS13, mean (SD)		13.45 (6.65)	15.24 (8.43)	12.57 (6.36)	16.97 (9.25)
ABETA42, mean (SD)		1180.82 (626.21)	1132.21 (627.38)	1249.41 (634.81)	1090.67 (607.52)
FDG, mean (SD)		1.27 (0.13)	1.25 (0.14)	1.28 (0.12)	1.23 (0.15)
APOE4 n (%)	0	731 (59.1)	817 (55.5)	405 (58.9)	1162 (54.1)
	1	414 (33.5)	524 (35.6)	236 (34.3)	780 (36.3)
	2	91 (7.4)	130 (8.8)	47 (6.8)	204 (9.5)
Hippocampus, mean (SD)		7039.70 (1105.52)	6888.08 (1190.04)	7193.61 (1071.92)	6794.01 (1185.75)
AV45, mean (SD)		1.17 (0.21)	1.17 (0.21)	1.16 (0.20)	1.21 (0.23)
ABETARatio, mean (SD)		0.14 (0.06)	0.14 (0.06)	0.15 (0.06)	0.13 (0.06)

Table 7 [Follow-up demographic and clinical characteristics of the ADNI cohort for $A\beta$ positive subjects for the three prediction tasks.]

		Change in diagnosis 2 years	Change MMSE 2 years	Change MMSE 4 years	Complete cohort at baseline
n		398	500	230	749
AGE, mean (SD)		74.00 (6.74)	74.06 (7.03)	73.45 (6.40)	73.67 (7.17)
gender n (%)	m	220 (55.3)	274 (54.8)	126 (54.8)	415 (55.4)
	f	178 (44.7)	226 (45.2)	104 (45.2)	334 (44.6)
MMSE, mean (SD)		27.79 (1.86)	26.97 (2.56)	27.96 (1.82)	26.55 (2.79)
ADAS13, mean (SD)		15.94 (7.07)	18.29 (8.73)	14.57 (6.62)	20.15 (9.57)
ABETA42, mean (SD)		756.52 (281.53)	729.31 (276.41)	773.47 (279.01)	753.97 (319.79)
FDG, mean (SD)		1.24 (0.13)	1.22 (0.14)	1.25 (0.12)	1.19 (0.15)
APOE4 n (%)	0	154 (38.7)	175 (35.0)	87 (37.8)	245 (34.5)
	1	180 (45.2)	238 (47.6)	106 (46.1)	345 (48.6)
	2	64 (16.1)	87 (17.4)	37 (16.1)	120 (16.9)
Hippocampus, mean (SD)		6767.78 (1024.99)	6602.03 (1105.63)	6967.73 (1001.39)	6517.64 (1090.63)
AV45, mean (SD)		1.35 (0.19)	1.36 (0.19)	1.34 (0.19)	1.37 (0.20)
ABETARatio, mean (SD)		0.09 (0.02)	0.09 (0.02)	0.09 (0.02)	0.09 (0.02)
